# Parkinsonian gait improvement through vibratory stride parameter feedback

**DOI:** 10.1186/s12984-026-02113-4

**Published:** 2026-08-01

**Authors:** Paul Kirsch, Miriam Helbig, Dominic Rupar, Simon Baudrexel, Christian A. Kell

**Affiliations:** 1 Translational Neuroscience at Cooperative Brain Imaging Center – CoBIC, Goethe University, Theodor Stern Kai 7, 60590 Frankfurt, Germany; 2 Department of Neurology, Goethe University, Theodor Stern Kai 7, 60590 Frankfurt, Germany

**Keywords:** Parkinson’s Disease, Cueing, Closed-loop feedback control, IMU, Walking

## Abstract

**Background:**

Hypokinetic gait increases morbidity and mortality of persons with Parkinson’s disease and constitutes a major target symptom for therapy. Feedback on gait quality, e.g. by a physiotherapist, improves stride length and reduces shuffling. Wearable devices measuring gait parameters and providing feedback could improve parkinsonian gait during everyday life when no physiotherapist is available. Here, we report on the efficacy of a new device providing discreet vibratory feedback upon decreased stride length and increased shuffling.

**Methods:**

33 persons with Parkinson’s disease, in two cohorts, received automatic vibratory feedback administered via a newly developed sensor-equipped insole when their stride length and heel strike angle decreased while walking a 730 m walking course. Gait of 14 persons with Parkinson’s disease in the first cohort was investigated in OFF and ON medication state to allow for a comparison of the feedback effect with the effect of medication on stride length, heel strike angle and gait variability. In both cohorts, the effect of feedback on gait parameters was compared against a control walk without stimulation and was subjectively evaluated by the study participants. In the second cohort of 19 participants, the effect of feedback was additionally compared with vibration at random time points to investigate the efficacy of context-adequate feedback.

**Results:**

Both stride length and heel strike angle improved upon feedback to a degree that, on average, was close to the medication effect on parkinsonian gait. Stimulation at random time points showed intermediate values with non-significant improvements in gait parameters compared to control walks. Closed-loop feedback resulted in a less variable gait pattern compared to control walks. Persons with Parkinson’s disease rated the feedback mode as subjectively useful.

**Conclusions:**

Our results demonstrate the use of closed-loop feedback can improve parkinsonian gait and suggest such a device could effectively complement the available approaches in the treatment of persons with Parkinson’s disease.

*Trial Registration:* This trial was retrospectively registered in the German Clinical Trials Register (DRKS00038516) on 9 December 2025*.*

**Supplementary Information:**

The online version contains supplementary material available at 10.1186/s12984-026-02113-4.

## Background

Gait dysfunction increases the mortality and hospitalization rate of persons with Parkinson’s disease (PwPD) [1–4] and reduces PwPD’s quality of life [5]. The typical hypokinetic-rigid parkinsonian gait is characterized by small stride lengths, shuffling, gait initiation and termination deficits, freezing, postural instability and falls. Besides pharmacotherapy, physiotherapy can improve gait considerably and thereby reduce the risk of falling [6]. Physiotherapists provide an external gait model and corrective commands in case they observe that PwPD's gait deteriorates, for example when stride length is reduced [7]. Given the limited availability of physiotherapists and the increasing prevalence of Parkinson’s disease (PD) [8], automated feedback (FB) on quantified gait parameters could provide an additional therapeutic option [9]. Such an approach is effective in laboratory settings, and improves gait more efficiently than open-loop cueing (for review, see [10, 11]).

FB has been shown to improve multiple gait parameters. Stride length and the heel strike angle (HSA) have consistently demonstrated responsiveness to FB interventions [12–18] and represent the two parameters most prominently discriminating healthy and parkinsonian gait already in early stages of the disease [19]. Both parameters describe distinct characteristics of the bradykinetic gait in PD. Stride length impairment is based on the reduced motion of the foot along its trajectory, while the deteriorated HSA results from a reduced range of motion in the ankle joint. Both parameters, that are in the focus of this study, are impaired in PD and clinically relevant, as they correlate both with risk of falls [3, 20], Hoehn and Yahr stage [21–23], and are responsive to medication intake [21, 24]. However, corrective commands can also reduce the variability of step cadence [25] and improve the stooped posture of PwPD through various FB modalities [16, 17, 26]. Visual FB based on the ground reaction force yields improvements beyond the propulsion force [27], thus suggesting that FB effects may generalize over gait parameters.

Various sensory modalities have been used previously for FB delivery [10]. While the auditory channel is often used for rhythmic auditory stimulation, it has also been used to provide FB via pre-recorded verbal instructions to change gait tempo [28]. Visual cues can also improve PwPD’s gait [29], but they can worsen posture [30] and both visual and auditory cues can distract PwPD’s attention from their environment [31, 32]. Vibratory stimuli have the advantage that they allow attending cues while keeping the audiovisual channels free for other tasks like communication, navigation, balance and posture. In addition, vibration via an insole is discreet and could thus reduce stigmatization compared to more obvious FB that also attracts attention of outside observers [33], especially as stigmatization reduces PwPD’s quality of life [34].

Here, we investigate the efficacy of a newly developed sensor-equipped insole that provides discreet vibratory feedback upon deterioration of stride length and HSA improving gait of PwPD walking a 730 m walking course. Most previous studies investigating gait in PD (for review, see [21]) and closed-loop cueing systems (for review, see [10]) use data collected on walkways in gait laboratories or on treadmills. This limits their transferability to everyday life [35, 36]. The inertial measurement unit (IMU) technology allows precisely measuring gait parameters also outside of laboratories [37] which could increase the ecological validity of investigations. However, investigating PwPD’s gait at home reduces comparability, because environments and mobility behavior differ [38] and the latter is even further influenced by home-based interventions [14]. We therefore investigated whether feedback on gait quality improved gait of PwPD walking a 730 m walking course consisting of everyday walking conditions. The inclusion of various walking contexts that participants could also encounter regularly in everyday life, resulted in a total length of the walking course of 730 m and an average walking duration of 12 min. Although this is longer than the typical everyday walking bouts of PwPD [36], longer walking bouts may unmask PD-related gait deterioration [38].

As our primary outcome measure, we analyzed feedback-related changes of stride length and HSA as well as the subjective evaluation of the FB algorithms by PwPD. To compare the FB effect with the medication effect, we investigated a first cohort of 15 PwPD (13 PwPD completed the full study protocol) both ON and OFF medication. The second cohort of 20 PwPD ON medication (19 PwPD completed the full study protocol) was investigated to replicate the results of the first cohort. Further, the second cohort also served to compare the FB algorithm with stimulation at random time points (random vibration: RV). This could clarify if the closed-loop algorithm itself was responsible for gait improvement or whether the recurrent reminder to improve gait was sufficient to elicit the effect. Post-hoc analyses included the FB effect on gait variability which is increased in parkinsonian gait [39] and is associated with falls [40] and disease progression [22]. Finally, the instantaneous FB effect was characterized by analyzing the five strides following each instance of FB.

## Methods

### Participants

Two cohorts of participants with gait disorder due to idiopathic PD (Hoehn and Yahr stage 1–3 [41]) were included in this study. Age, H&Y stage, Levodopa Equivalent Dose [42], disease duration, cognitive capacities as objectified in the PANDAs-test [43] and scores of the Motor Examination (Part III) of the MDS-UPDRS [44] are reported in Table 1 and Supplementary Table 1. The postural instability and gait difficulty score was defined by the sum of the MDS-UPDRS Part III subcores of gait, postural stability and freezing similar to [45] and is provided in Supplementary Table 1. PwPD with pallanaesthesia of the feet or ankles as stated in previous medical records and examined with a tuning fork (< 6/8), cognitive impairment as objectified in the PANDAs-test [43], dependence on a wheelchair or a walker, and gait disorders other than PD were not included in this study. Participants were recruited from the Neurological outpatient department of Goethe University Hospital and from self-help groups. All participants were unpaid.

**Table 1:** Participants’ characteristics (IQR: interquartile range, H&Y: Hoehn and Yahr): Characteristics did not differ significantly between cohorts as determined by a Wilcoxon-Test for continuous variables and Fisher's-exact-test for categorical variables

	Median (IQR)—Cohort 1	Median (IQR)—Cohort 2	p-value
Age	59 (6.5)	61 (3)	0.124
Gender	11 Male, 4 Female	14 Male, 6 Female	1
H&Y stage	3 × I, 6 × II, 6 × III	4 × I, 12 × II, 4 × III	0.228
Levodopa Equivalent Dose	548 (171.5)	418 (323)	0.308
Years since first diagnosis	5.5 (5)	5 (5.5)	0.36
PANDAS score	23 (6.8)	22 (4.5)	0.912
UPDRS 3 score (ON medication)	25 (6)	30 (21.5)	0.551

**Fig. 1:**
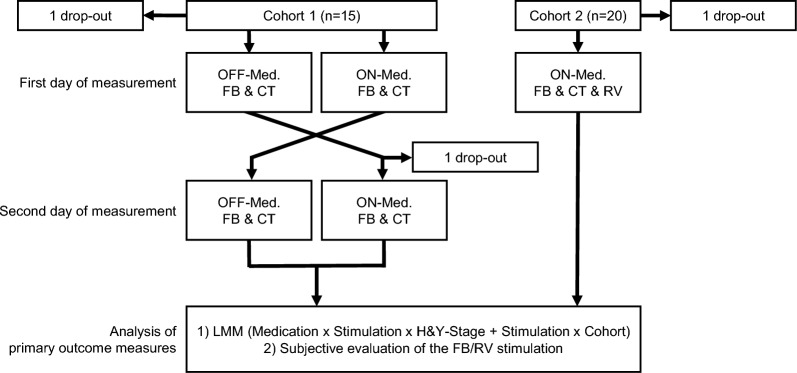
Study design. Cohort 1 consisted of PwPD participating both ON and OFF medication (Med.) on separate days in a counterbalanced order. Within each day of measurement, PwPD walked once with feedback (FB) and once without feedback (control: CT) in counterbalanced order. PwPD in cohort 2 participated in ON medication state in FB and CT walks as well as walks with vibration at random time points (RV) in a counterbalanced order. Primary outcome measures included average stride length and heel strike angle of both cohorts and were analyzed in linear mixed models (LMM). The subjective evaluation of the FB/RV stimulation by the PwPD was analyzed using T-tests

The first cohort of fifteen PwPD (see Table 1) served to investigate the effect of closed-loop feedback on parkinsonian gait and to compare this effect with the medication effect by comparing PwPD when they were ON their regular medication and when they were OFF medication. PwPD thus came in on two occasions, the order of medication states was counterbalanced. We expected a deterioration of gait parameters when comparing control walks in the OFF and ON medication conditions and investigated whether gait parameter FB resulted in improved gait. The second cohort of twenty PwPD (see Table 1) was measured solely ON medication and served to replicate the FB effect in an independent PwPD sample and to further investigate the benefit of closed loop stimulation vs. stimulation at random time points. The study design is depicted in Fig. 1.

The time interval between the ON and OFF medication measurements was 22 (standard deviation (SD) = 12) days and PwPD were measured at the same time of day on both occasions. OFF measurements were recorded in the morning after overnight withdrawal from all PD medications. Due to work commitments, one participant was recorded in the evening after receiving only a very early morning dose followed by drug withdrawal during the day. After their first OFF medication recording, one participant was not available anymore for the ON medication measurement. Another PwPD stopped participating in this study after a first control walk due to exhaustion, leaving 13 PwPD with full datasets in cohort 1 (see Supplementary Table 2 for details). In cohort 2, one PwPD dropped out because they felt exhausted after a first measurement, leaving 19 full datasets for analysis (see Supplementary Table 2 for details).

### Insole and feedback

**Fig. 2:**
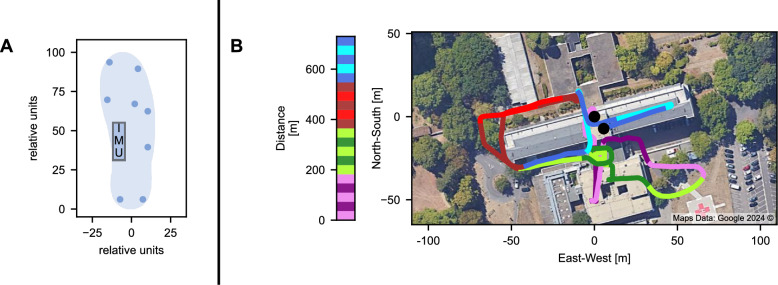
Set-up of the sensors in the insole and the walking course. **A** Layout of a right insole with the coordinates of the eight force sensors (blue dots) and the IMU (blue square) **B** Exemplary coordinates calculated from the IMU-measured acceleration data with start and end (black dots) of the walking course. Color indicates the distance covered

Two insoles equipped with an IMU and eight pressure sensors (Fig. 2A) were connected to a microcontroller that monitored PwPD’s gait at 100 Hz (prototype developed by Novapace, Darmstadt, Germany). Three different sole sizes assured good individual fit. The IMU’s data on axial and rotational acceleration were online filtered using a C +  + implementation of a Madgwick-Orientation-Filter [46] to weigh axial and rotational acceleration differently depending on the degree of acceleration. The coordinates of the IMU were calculated by integrating the acceleration. The IMU coordinates were used to calculate stride length and HSA. Stride length was defined as the distance travelled during one swing phase. HSA was approximated as the maximal pitch angle of the IMU describing the maximal dorsalextension of the foot. The maximal pitch angle occurred on average 40 ms around the first sensor registration of ground reaction force of a stance phase.

Gait throughout the entire 730 m walking course was monitored by evaluating stride length and HSA to provide feedback on gait quality. A rule-based algorithm was implemented to detect gait deterioration based on stride length and HSA. For each current step, the values of both parameters of the eight preceding steps were retained to enable an analysis across a sliding window. When both parameters decreased multiple times between two successive strides within the last eight strides (i.e., 10 of the 14 stride-to-stride changes), FB on the respective sole was triggered once within the stance phase following the last heel strike. A continuous decrease of just one of the two parameters was thus not sufficient to trigger feedback. FB was also not applied in case of outlier values or during voluntary braking [47–49]. A refractory period of 20 s was implemented to prevent overstimulation. This FB algorithm proved to be the most reliable out of multiple tested algorithms in 15 additional PwPD investigated during prototype development.

Vibratory feedback consisted of vibrotactile stimulation via a vibrating actor built in the insole vibrating at 200 Hz for 0.2 s. Because gait can be asymmetrically affected by PD [21], each foot received its proper feedback separately. Before entering the walking course, participants received a standardized instruction from PK to react to the vibrotactile feedback by making larger stride cycles and to reduce dragging their feet. Individual follow-up questions were answered as needed.

In cohort 2, vibration was delivered either within the described closed-loop paradigm or randomly while the instructed reaction to stimulation was kept constant. To control the overall amount of stimulation events, RV was applied at random time points between 7 and 20 s after gait deterioration that in the closed-loop condition would have triggered direct feedback. In 50% of walks with RV, an additional stimulation was provided to account for possible missed events at the end of the walk. RV stimulation was not applied at completely random time points, because the number of RVs would have needed to be determined in advance and could differ from the number of FB stimulations in the same participant.

Participants were not informed about the condition they were in and were instructed to react to stimulation regardless of the context in which stimulation occurred. The average number of stimulations per minute was 1.54 in the FB condition, 1.55 in the RV condition, and the number of time points suited for FB in walks without stimulation was 1.53 (see Conditions). FB was triggered while PwPD walked straight (59.5% of feedback signals occurred in this walking situation) or in curves (19.4%), during up- or downhill walking (12.8%) and when PwPD accelerated (5.3%), decelerated (3.0%). Classification into those categories was based on the video-label (see Off-line statistics and analysis).

The subjective usefulness of the FB device was evaluated by PwPD after each walking course, by indicating how often they would use the applied stimulation in everyday life on a visual analog scale (VAS) from 1 to 10 (1: “I would never use the device”, 5: regularly, and 10 always).

### Conditions

In the first cohort, every measurement consisted of one walk without stimulation (control, CT) and one with FB, with 5-min pauses in between. A measurement in the second cohort consisted of a recording of one walk without stimulation (CT), one with FB and one with RV, with 5-min pauses in between. The order of conditions in both cohorts was counterbalanced, to reduce the effect of learning, habituation and fatigue. The mixed-model analysis included the order as a fixed effect to account for its effect. PwPD were blind regarding the stimulation mode. The study design is depicted in Fig. 1.

### Naturalistic walk

The walking course (Fig. 2B) consisted of a 730 m walk (average walking duration 12.8 min; SD = 1.6 min) through the university hospital and its surroundings with PK filming the PwPD from behind. 50% of the walking course lead through the hallways of the hospital, 10% were located on a parking lot and 40% on a sidewalk. Together with experienced physiotherapists, the path was chosen to include everyday situations with narrow spaces, ramps, staircases and crowded areas.

### Off-line analyses

The video recording was used to classify each step as belonging to one out of 18 walking situations (see Supplementary Table 3). Off-line analyses of gait parameters were performed on strides during straight forward walking, which is the classical condition in cueing studies on walkways [10], and strides during curve walking, which can exacerbate parkinsonian gait disturbances [50].

The IMU’s data were exported using an SD card. The acceleration data were fed to the same Madgwick-Orientation-Filter as used on the microcontroller [46], however in a python environment (imufusion package). Stride length, stride duration (duration of the swing phase; used to exclude multiple stride cycles marked as one) and HSA were calculated the same way as on the microcontroller. Comparisons between online and offline values showed identical results. Strides with the situation labels of straight forward walking, accelerating, decelerating, left curve and right curve walking (see Supplementary Table 3) were included. Then, the overall distributions of stride length and stride durations (total number of strides = 104,871) were examined and strides in the beginning of acceleration and the end of deceleration phases (defined as stride length < 0.75 m or stride duration < 0.6 s: n = 2156) and multiple stride cycles marked as one (defined with stride duration > 1.4 s or stride length > 2 m: n = 141) were excluded. This resulted in the inclusion of on average 950 (SD = 114) strides per walk.

### Statistics

#### Primary outcome measure: effect of FB on stride length and HSA

We investigated the FB and RV effect on stride length and HSA in two linear mixed models (LMM) that took the interactions between FB and H&Y stage or medication state or cohort into account. These models explaining averaged stride length and HSA per participant thus included stimulation mode, medication state, H&Y stage, number of cohort, order of walk within a measurement, number of measurement day, and—for the LMM of stride length—body height as main effects with interactions between H&Y stage, stimulation mode and medication state as well as an interaction between cohort and stimulation mode. The formula was: HSA / stride length ~ Stimulation mode * Medication * H&Y + Stimulation mode * Cohort + Body height + Number of walk + Number of measurement day + (1| PwPD). Post-hoc testing was conducted using marginal contrasts [51] and the Cohen’s d for paired data was calculated using the function “eff_size” of the R package “emmeans”.

#### Primary outcome measure: subjective evaluation of the FB/RV stimulation by PwPD

Participants indicated directly after each FB and RV walk how often they would use such a stimulation device in everyday life. The answers were compared between FB and RV walks and between medication states using paired two-sided T-Tests (python package “scipy”), and between cohorts using an unpaired two-sided T-Test (python package “scipy”). Data were tested for non-normal distribution using the Shapiro–Wilk-Test.

#### Post hoc* analysis: effect of stimulation on gait variability*

The variability in stride length and HSA was investigated by calculating the parameters’ coefficient of variation (COV). To reduce the effect of time during the course (e.g. related with exhaustion during the 13 min walk), dispersion measures of stride length and HSA were calculated using the COV (= 100*Standard Deviation / Mean) of each fraction of 5% (≈ 36 m) of the walking course in fractions with at least 10 strides labeled as straight forward walking, de- and accelerating [52]. Curve walking was not included in the COV analyses because it is naturally asymmetric and could thus interfere with variance analyses. Strides in the beginning of acceleration and the end of deceleration phases (defined as stride length < 0.75 m or stride duration < 0.6 s) and multiple stride cycles marked as one (defined with stride duration > 1.4 s or stride length > 2 m) were excluded, because the smaller strides could impede the estimation of the COV [52]. This resulted in the inclusion of on average 704 (SD = 109) strides per walk and on average 16.4 (SD = 2.11) fractions per walk and side (mean number of strides per fraction = 21.7, SD = 5.4). The data in this analysis were logarithmically transformed to obtain a normal distribution of the residuals. An LMM with the following formula: log(COV) ~ Stimulation mode * Medication * H&Y + Stimulation mode * Cohort + Number of walk + Number of measurement day + (1| PwPD) was fitted to investigate main effects of medication state and stimulation mode and interactions of medication state and stimulation mode with H&Y stage. Post-hoc testing was conducted using marginal contrasts [51].

#### Post hoc* analysis: stride-resolved characterization of the stimulation effect*

The instantaneous effect of FB on stride length and HSA was assessed by analyzing the changes between subsequent strides in stride length and HSA. Some PwPD received more FB than others, resulting in an unbalanced number of FB occurrences to be analyzed. To include every FB occasion in the analysis and not average per PwPD beforehand, stride length and HSA were rendered comparable between PwPD by z-scoring within each medication state of each PwPD over all conditions. The analyzed segments were then defined by selecting the stride during which stimulation occurred (or in CT walks, the stride at which the FB algorithm would have elicited feedback) as the respective stride 0. Figure 4 depicts the 8 strides before and the 5 strides after stride 0. The effect of stimulation was evaluated in the five strides after stride 0, in which the differences in step length or HSA between successive strides (first order difference: stride_n_ – stride_n+1_) were calculated. The first order differences in FB and CT walks were compared using an independent two-sided T-test (python package “scipy”). RV was investigated by comparing the first order differences to 0 using a two-sided one sample T-Test (python package “scipy”). Data distributions were evaluated by visual inspection [53]. P-values of the five first order differences after stride 0 were Bonferroni-corrected.

LMMs were implemented using the package “lmertest” where degrees of freedom were estimated with the Satterthwaite’s method and post-hoc testing was done using marginal means [51] from the package “emmeans” and “modelbased” using Z-tests. Unless denoted otherwise, coefficients concerning stride length are denoted in centimeters and coefficients describing HSA in degrees. All statistical tests assumed significance at p < 0.05.

## Results

### Primary outcome measure: effect of FB on stride length and HSA

**Fig. 3:**
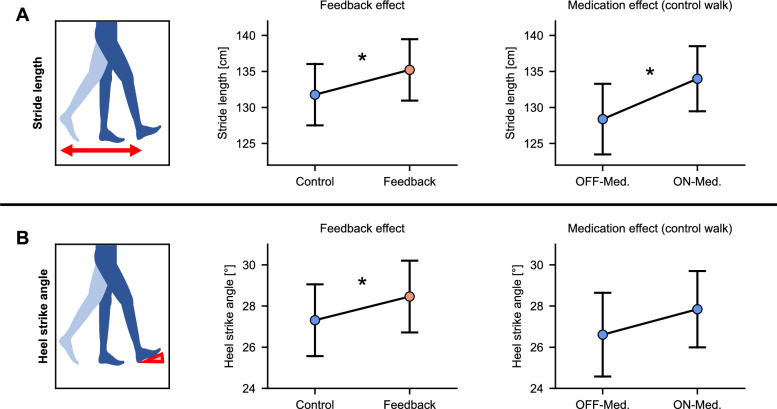
Feedback and medication improve average stride length (**A**) and heel strike angle (HSA; **B**). The middle panels illustrate the estimated means of stride length / heel strike angle in both control (blue) and feedback (orange) condition. The right panels illustrate the estimated means of both parameters in ON and OFF medication during the control walk (blue) for comparison with the FB effect. Estimated means account for between-subject variability, the errorbars illustrate 95%-confidence intervals or the estimated means. Increased values indicate improvement, decreased values deterioration of gait parameters. * indicates significance at p < 0.05. For the influence of medication, Hoehn and Yahr stage and cohort on the FB effect, please see Supplementary Fig. 1

Both stride length and HSA increased upon FB compared to CT (Fig. 3A; main effect of FB on stride length: difference = 3.44, p < 0.001, Z = 4.2, Cohen’s d = 0.202; main effect of FB on HSA: difference = 1.15, p = 0.001, Z = 3.18, Cohen’s d = 0.164). Medication led to an increase in stride length (main effect of medication: difference = 5.42, Z = 3.49, p < 0.001, Cohen’s d = 0.318) but not significantly in HSA (main effect of medication: difference = 1.07, p = 0.117, Z = 1.57, Cohen’s d = 0.153). The coefficient of the FB-related increase in stride length amounted to 63% of the medication effect. The coefficient of the FB-related increase in HSA amounted to 107% of the medication effect.

### Effect of RV on stride length and HSA

RV walks showed intermediate values between CT and FB walks which neither differed significantly from CT walks (stride length: difference = 2.38, p = 0.107, Z = 1.61, Cohen’s d = 0.158; HSA: difference = 1.06, p = 0.068, Z = 1.83, Cohen’s d = 0.128, not illustrated) nor from FB walks (stride length: difference = 0.3, p = 0.842, Z = -0.2, Cohen’s d = 0.037; HSA: difference = 0.16, p = 0.78, Z = 0.28, Cohen’s d = 0.024, not illustrated).

### Interactions of the FB effect with H&Y -stage, medication state and cohort

The FB effect was consistent across medication states, H&Y-stage and cohorts. There was no significant interaction between the medication and the feedback coefficient in both stride length (estimate = 0.077, p = 0.972, Z = 0.036, see Supplementary Fig. 1, upper panel) and HSA (estimate = 0.349, p = 0.714, Z = 0.367). Moreover, there was no significant interaction between cohort and FB effect (see Supplementary Fig. 1, lower panel), in both stride length (estimate = 1.83, p = 0.367, Z = 0.902) and HSA (estimate = 0.907, p = 0.311, Z = 1.01), indicating replication of the FB effect in the second cohort.

PwPD of H&Y stage 1 showed an increased stride length when compared with H&Y stage 2 (difference = 14.2, p = 0.018, Z = 2.37, Cohen’s d = 1.19) and H&Y stage 3 (difference = 22.3, p < 0.001, Z = 3.50, Cohen’s d = 1.87). HSA was increased in PwPD of H&Y stage 1 when compared to H&Y stage 2 (difference = 4.66, p = 0.043, Z = 2.02, Cohen’s d = 0.942) and H&Y stage 3 (difference = 12.8, p < 0.001, Z = 4.83, Cohen’s d = 2.58), as well in H&Y stage 2 when compared to H&Y stage 3 (difference = 8.11, p < 0.001, Z = 3.90, Cohen’s d = 1.63). There was no significant interaction between the H&Y stages and the FB effect (see Supplementary Table 4 and 5 and Supplementary Fig. 1, middle panel).

### Primary outcome measure: subjective evaluation of the FB/RV stimulation by PwPD

PwPD rated the stimulation as overall useful, with no significant differences in ratings across cohorts, medication states, or feedback modality. PwPD were asked to indicate how often they would use the applied stimulation in everyday life on a visual analog scale (VAS) from 1 to 10 (1: “I would never use the device”, 5: regularly, and 10 always). On average, the first cohort rated the usefulness of FB both OFF and ON medication 7.2 (SD = 2.3), the second cohort rated FB with 5.8 (SD = 1.7) and RV with 5.6 (SD = 2.1). PwPD’s evaluation neither differed significantly between cohorts (independent sample T-test: p = 0.089, T(30) = 1.76), medication states (dependent sample T-test: p = 0.337, T(12) = 1) nor between RV and FB (dependent sample T-test: p = 0.298, T(18) = 1.07).

### Post hoc* analysis: effect of stimulation on gait variability*

FB did not improve the log(COV) of stride length significantly but decreased the variability of the HSA (Stride length: estimate = 0.004, p = 0.848, Z = 0.19, Cohen’s d = 0.02; HSA: estimate = 0.06, p = 0.033, Z = 2.13, Cohen’s d = 0.166). Gait variability did neither differ significantly between RV or CT (Stride length: estimate = 0.02, p = 0.67, Z = 0.43, Cohen’s d = 0.071; HSA: estimate = 0.04, p = 0.423, Z = 0.8, Cohen’s d = 0.101) nor between RV and FB walks (Stride length: estimate = 0.06, p = 0.123, Z = 1.54, Cohen’s d = 0.26; HSA: estimate = 0.05, p = 0.343, Z = 0.95, Cohen’s d = 0.121). The log(COV) of stride length but not the log(COV) of HSA improved significantly with medication intake in CT walks (Stride length: estimate = 0.11, p = 0.013, Z = 2.48, Cohen’s d = 0.485; HSA: estimate = 0.11, p = 0.058, Z = 1.9, Cohen’s d = 0.281). PwPD in H&Y stage 3 had a more variable gait than PwPD in H&Y stage 1 (Stride length: estimate = 0.24, p = 0.002, Z = 3.11, Cohen’s d = 1.56; HSA: estimate = 0.59, p < 0.001, Z = 4.15, Cohen’s d = 2.17) and in H&Y stage 2 (Stride length: estimate = 0.14, p = 0.022, Z = 2.3, Cohen’s d = 0.915; HSA: estimate = 0.41, p < 0.001, Z = 3.61, Cohen’s d = 1.48). For further results of the LMMs see Supplementary Table 6 and 7 and Supplementary Fig. 2.

### Post hoc* analysis: stride-resolved characterization of the stimulation effect*

**Fig. 4:**
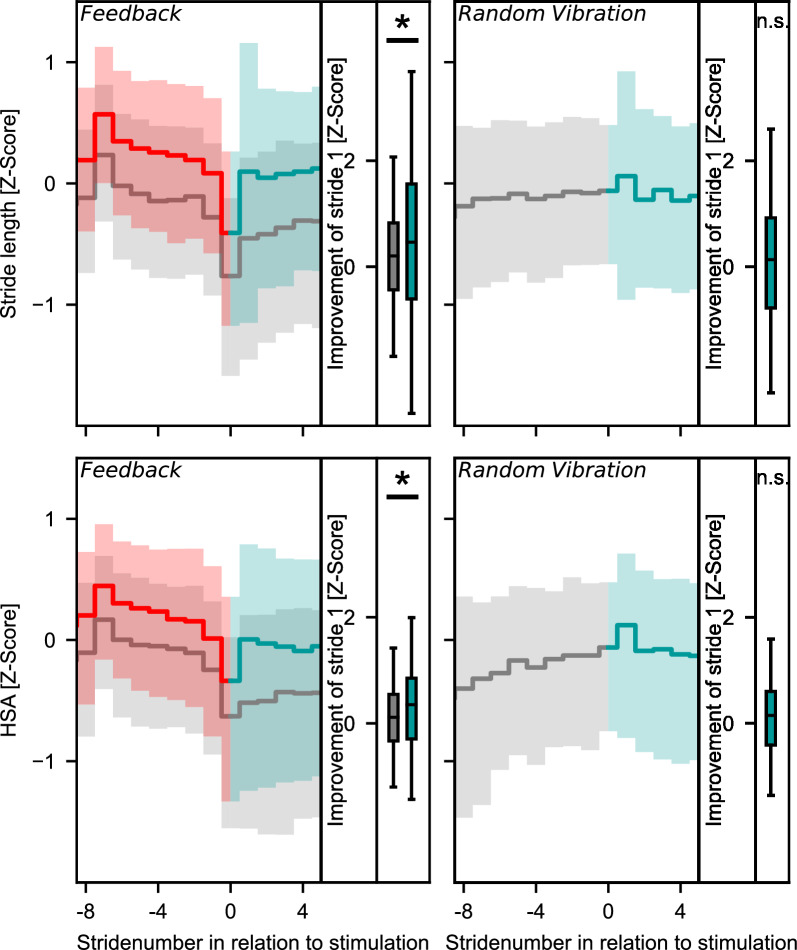
Feedback instantaneously improves stride length and heel strike angle. Stride-resolved median Z-Score (within each PwPD & medication state) of stride length (upper panels) and heel strike angle (HSA, lower panels) before and up to 5 strides after stimulation. Left panels illustrate reactions to feedback (FB; red: strides before stimulation, green: strides after stimulation) compared to control (CT) walks (grey: strides at which a vibration would have been triggered during a FB walk), right panels illustrate strides before (grey) and after stimulation at random time points (RV; green). Shaded areas depict the interquartile range. On the right of each panel: Improvement (first order difference between stride 0 and stride 1) upon stimulation in the FB walk (green) or at similar strides of CT walks (grey) when FB would have occurred in the FB run. The improvement upon RV (green in right part of the right panels) is tested against 0. * indicates significance at p < 0.05; n.s.: not significant

Closed-loop FB improved gait directly after deterioration. The change (first order difference) in stride length and HSA between the stride during which FB was administered and the first stride after FB was larger than the change between a stride during a CT walk that would have triggered stimulation in a FB walk and its successive stride (independent sample T-tests with Bonferroni correction; stride length: p_corr_ = 0.023, T(2748) = 2.83; HSA: p_corr_ < 0.001, T(2748) = 4.52; Fig. 4). The first order difference of the following strides did not differ significantly anymore between FB and CT walks, suggesting that PwPD did not improve their gait promptly even at later strides after gait deterioration in CT walks. RV neither increased stride length significantly (one sample T-test with Bonferroni correction: p_corr_ = 1, T(619) = 1.21) nor did it increase HSA significantly (one sample T-test p_corr_ = 0.415, T(619) = 1.74) in the first stride after RV (Fig. 4).

## Discussion

This study demonstrated a gait improvement in PD using haptic FB delivered by a new device that signals deterioration of gait parameters in a 730 m walking course that contained numerous everyday situations. The effect of FB on both stride length and shuffling as evidenced by the marginal means of the model output showed an improvement that was close to the medication effect in the same model. Improvements occurred instantaneously after FB administration. PwPD indicated that they would use device-assisted FB frequently. PwPD's gait became less variable, which has been related with reduced risk of falls [40]. Together, these results indicate the usefulness of such a new device in treating PD. The central new addition of this device to the therapy of PwPD is the discreet availability of gait-improving FB in everyday life. It can thus extend benefits from physiotherapy and can help maintain a less variable gait which could reduce the risk of falls.

### Primary outcome measure: effect of FB on stride length and HSA

FB-related gait improvement was of a small effect size. In previous studies investigating FB on stride length or the HSA, standardized effect sizes were reported seldomly. In treadmill studies, the effect of FB on stride length showed a small effect in the best-performing trials of PwPD (Cohen’s d of 0.23) [27], and a moderate effect on the HSA (Cohen’s d of 0.56) [17]. The scarcity of standardized effect sizes limit cross-study comparisons of the effects in this study, especially as the mentioned effect sizes stem from treadmill walking and are thus not directly transferable to overground walking conditions [54]. Our study design permitted to compare the FB with the medication effect on gait. Like the FB effect, the medication effect was similarly small, and the FB effect amounted to 63% of the medication effect for stride length and 107% for HSA. In the absence of applicable minimal clinically important differences (MCIDs) for stride length and heel strike angle (cw. [22]), we interpret the FB effect as clinically relevant, as the effect of the standard medication would obviously be considered the currently best achievable therapy effect in the studied PwPD. Whether the FB mode of the current device indeed reduces falls has to be determined in future studies with longer observation periods in PwPD’s everyday life.

### Primary outcome measure: subjective evaluation of the FB/RV stimulation by PwPD

PwPD judged both FB and RV stimulation as useful, which suggests that PwPD were not aware of the additional benefit of closed- vs. open-loop stimulation. Overall, this observation confirms previous negative reports on PwPD’s attitudes towards verum and high-level control conditions [55] and could result from the motivational effect of exercise and feedback in itself [56]. The subjective usefulness could also result from the fact that the studied device was more discreet than previously described set-ups [10]. The inclusion of RV in the second cohort could have affected the face validity of FB, which could explain slightly lower usefulness ratings of FB in the second cohort. This can be regarded as a limitation of our study design.

### Interactions of the FB effect with H&Y-stage, medication state and cohort

Previous intervention studies showed that cueing and FB effects differed between differently affected PwPD and largest effects were found in intermediately affected PwPD, with sufficient capacities to process and respond to FB or cueing on gait [57–59]. This could be due to moderately impaired PwPD having a high potential for improvement but more capacities to implement improvements than late-stage PwPD. In this study, the FB effect generalized over medication states as well as disease stages. However, due to the small subgroups, the lack of significant interactions between FB effects and medication state as well as disease progression, need to be interpreted with caution and could be responsible for the interindividual variation of the FB effect. Further, the variability in the FB effect could be due to factors like the order of conditions and individual variability in the response to external stimuli like outside walking [60].

Generalizability of the FB effect was suggested by an absence of a significant cohort*FB interaction.

### Effect of RV on stride length and HSA

In contrast to most previous studies (for review, see [10]) we also compared closed-loop FB directly with stimulation at random time points. Because the instructions on how to react to stimulation were kept constant, the only difference lay in the time point of FB and thereby addressed the question, whether closed-loop FB is more useful than arbitrary reminding PwPD to maintain their gait performance in an open-loop manner. RV produced overall intermediate effects between walking without stimulation and walking with closed-loop FB. The insignificant improvement in the RV condition and the improvement in the FB condition may be attributable, at least in part, to PwPD paying increased attention to their gait performance in walks with vibratory stimulation. In that case, RV could have also improved walking significantly because the instruction to react was the same. However, the PwPD used cues better in plausible situations in which they could reasonably react, because stimulation occurred in the correct context (i.e. after worsened gait), which resulted in an FB-related improvement of averaged gait parameters.

### Post hoc* analysis: effect of stimulation on gait variability*

FB decreased the variability of the HSA indirectly, because the initial instruction how to react to FB did not include any reference to variability. Closed-loop FB terminated periods of transient gait deterioration in the FB condition quickly, which reduced overall HSA variability. However, additional carry-over effects of FB on gait variability cannot be excluded. This contrasts with external open-loop cueing, which has been shown to deteriorate gait variability as an adverse effect, while improving other gait parameters [61].

### Post hoc* analysis: stride-resolved characterization of the stimulation effect*

In contrast to RV walks, FB at the contextually adequate time point led to an immediate increase of both stride length and HSA. Hence, FB could provide external cues that substitute faulty internal models [10], while RV did not achieve this in our study. This suggests that the immediate reaction to FB observed during 730 m-long walks could in principle also benefit PwPD during shorter bouts of walking, that are typically observed during their everyday life [36]. This has to be investigated in future studies.

### Limitations

Although the device is supposed to be used for longer periods of time, we investigated about 26 min of FB from each PwPD in the first cohort and 13 min of FB from each PwPD in the second cohort. We expect the usefulness of FB to generalize to longer device use, but this has yet to be proven empirically. However, the efficacy of the device could diminish over longer periods of use, as could the FB effect, although the salient closed-loop FB may habituate less compared to open-loop stimulation [10].

Attending the FB for longer periods could in theory lead to fatigue and require prolonged attention to the stimulation, although the stimulation is salient. Nevertheless, a previous study showed that FB compared to walking without stimulation does not lead to an increased fatigue in 30 min walks [28]. Further, PwPD may use salient FB as attentional cues to improve their walking in dual-task conditions [62, 63], where cues facilitate directed attention towards gait [64]. This is particularly important, because gait deterioration in dual-tasks is often the most safety–critical aspect of mobility in PwPD [65]. Moreover, it has been hypothesized that cueing may even decrease the attentional cost of walking, as it reduces the cognitive resources PwPD must direct toward gait monitoring [66].

Studying the long-term use in PwPD’s everyday life will permit investigating the FB effect without an accompanying observer, preventing a Hawthorne observer effect [67], and allow studying any potential impact on falls. As gait impairments in PD include also other aspects beyond stride length and HSA [21], individual FB algorithms tailored to each PwPD’s individual symptoms could improve the effect of FB even further. Even though long walking bouts unmask gait deterioration in PwPD [38], FB could also be applied after shorter sequences of gait deterioration, that could prove useful particularly during short walking bouts such in at-home-environments [36].

## Conclusions

Taken together, we report the efficacy of a new discreet device that provides vibratory FB on gait parameters and improves hypokinesia, reduces shuffling and decreases gait variability in PwPD largely independent of medication state and disease progression. Such a device could complement therapeutic efforts in PwPD, reduce falls and improve quality of life.

## Supplementary Information

Supplementary file 1.

Supplementary file 2.

Supplementary file 3.

## Data Availability

The dataset supporting the conclusions of this article is available in the figshare repository, [10.6084/m9.figshare.30774530].

## Abbreviations

PD: Parkinson’s disease
PwPD: Person with Parkinson’s disease
HSA: Heel strike angle
FB: Feedback
RV: Stimulation at random time points (randomly timed vibration)
CT: Control
COV: Coefficient of Variation
