# Supplementary material for: Parkinsonian gait improvement through vibratory stride parameter feedback
Source: J Neuroeng Rehabil. 2026 Aug 1;23:226. doi: 10.1186/s12984-026-02113-4 (PMC13430838; doi:10.1186/s12984-026-02113-4)
Supplement: Supplementary file 1 [file 12984_2026_2113_MOESM1_ESM.pdf]

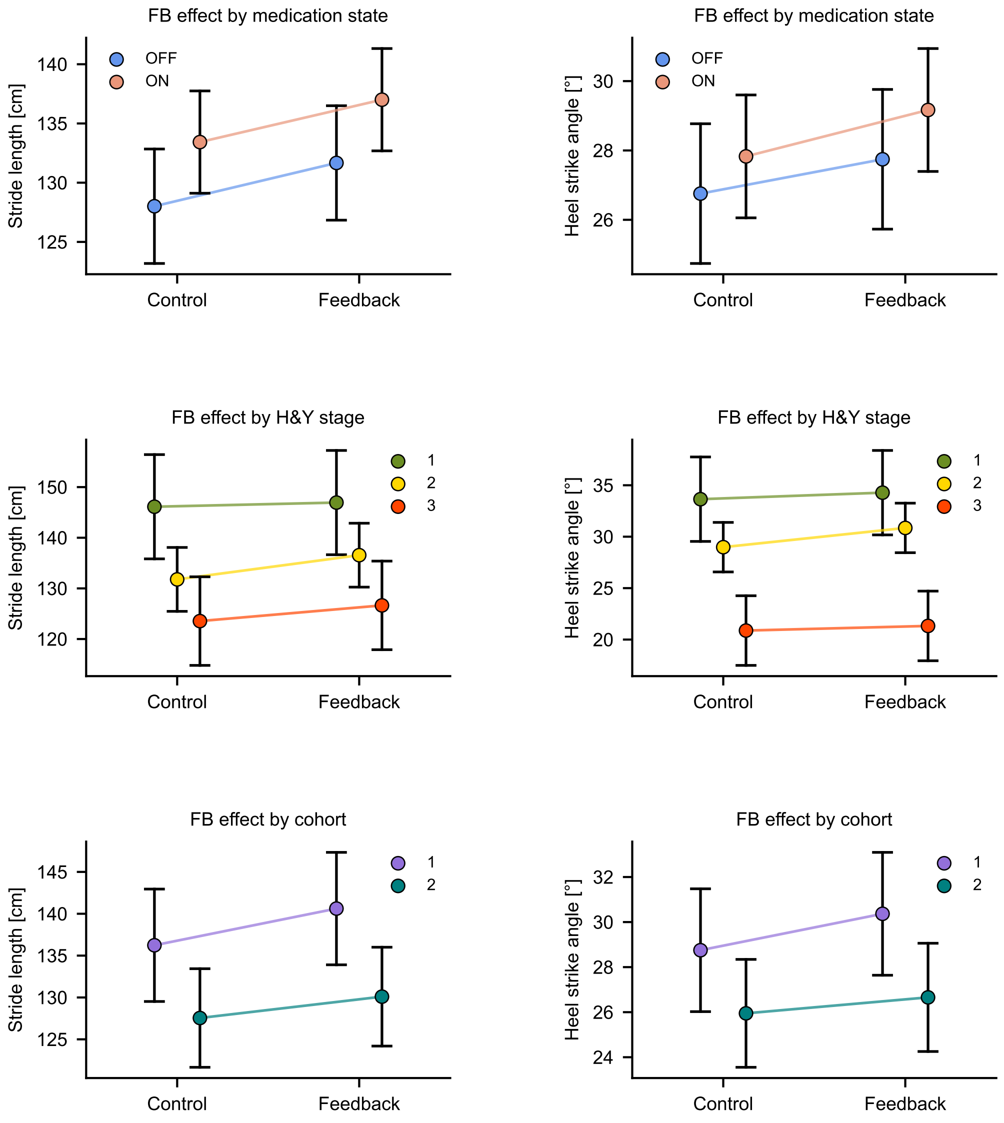

FB effect by medication state
Stride length [cm]
OFF
ON
Control
Feedback
FB effect by medication state
Heel strike angle [°]
OFF
ON
Control
Feedback
FB effect by H&Y stage
Stride length [cm]
1
2
3
Control
Feedback
FB effect by H&Y stage
Heel strike angle [°]
1
2
3
Control
Feedback
FB effect by cohort
Stride length [cm]
1
2
Control
Feedback
FB effect by cohort
Heel strike angle [°]
1
2
Control
Feedback
