# Supplementary material for: Parkinsonian gait improvement through vibratory stride parameter feedback
Source: J Neuroeng Rehabil. 2026 Aug 1;23:226. doi: 10.1186/s12984-026-02113-4 (PMC13430838; doi:10.1186/s12984-026-02113-4)
Supplement: Supplementary file 2 [file 12984_2026_2113_MOESM2_ESM.pdf]

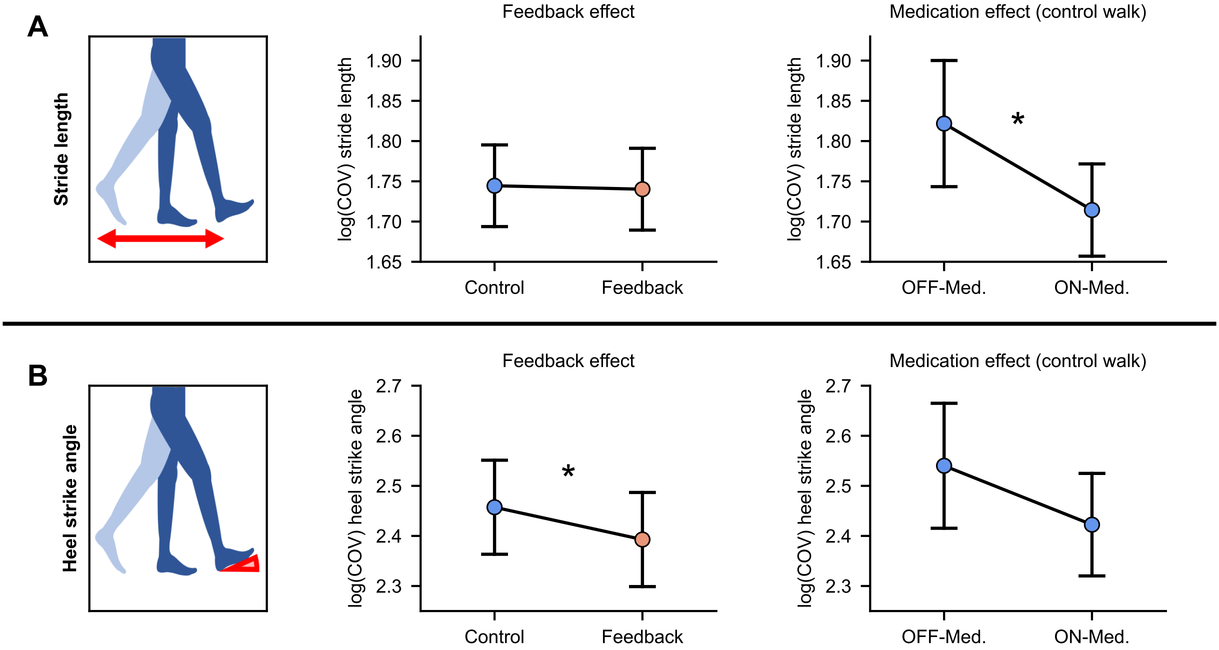
A
Stride length
Feedback effect
log(COV) stride length
1.90
1.85
1.80
1.75
1.70
1.65
Control
Feedback
Medication effect (control walk)
log(COV) stride length
1.90
1.85
1.80
1.75
1.70
1.65
*
OFF-Med.
ON-Med.
B
Heel strike angle
Feedback effect
log(COV) heel strike angle
2.7
2.6
2.5
2.4
2.3
*
Control
Feedback
Medication effect (control walk)
log(COV) heel strike angle
2.7
2.6
2.5
2.4
2.3
OFF-Med.
ON-Med.
