# Supplementary material for: Parkinsonian gait improvement through vibratory stride parameter feedback
Source: J Neuroeng Rehabil. 2026 Aug 1;23:226. doi: 10.1186/s12984-026-02113-4 (PMC13430838; doi:10.1186/s12984-026-02113-4)
Supplement: Supplementary file 3 [file 12984_2026_2113_MOESM3_ESM.pdf]

Supplementary Table 1: Patients' characteristics

Participant ID[1]	Age [years]	Sex [M/W]	H&Y	LED [mg]	Height [cm]	Time since diagnosis [years]	Pandas	UPDRS 3 ON Med.	UPDRS 3 OFF Med.	PIGDs-Score[2] ON Med.	PIGDs-Score[2] OFF Med.
P2_06	48	W	1	300	164	4	27	25	38	1	3
P2_12	60	M	1	200	180	2	26	33	32	2	2
P2_02	58	M	2	656	174	4	23	28	29	1	2
P2_07	49	M	2	566	185	8	18	39	40	3	3
P2_08	58	M	2	300	181	16	18	25	22	1	2
P2_09	64	M	2	542	178	3	19	30	43	1	3
P2_11	59	M	2	750	187	20	25	25	37	1	4
P2_15	59	M	2	610	176	15	23	15	32	1	3
P2_03	74	M	3	1000	178	9	25	29	34	6	6
P2_04	73	W	3	450	160	6	18	29	38	5	6
P2_05	55	M	3	420	178	5	19	22	39	3	6
P2_10	56	W	3	560	172	4	26	23	28	2	3
P2_14	60	M	3	554	173	9	25	22	25	2	3
P2_01	67	W	3	530	170	2	18		55		5
P3_01	64	W	1	0	170	1	21	13		1	
P3_09	60	M	1	0	182	3	24	42		2	
P3_15	59	M	1	405	179	0	19	23		1	
P3_20	71	W	1	565	172	1	24	13		2	
P3_03	43	M	2	60	179	6	23	24		3	
P3_04	59	M	2	470	183	5	26	30		3	
P3_05	74	M	2	450	174	4	18	30		3	
P3_06	61	M	2	683	181	5	20	78		2	
P3_08	73	M	2	238	183	2	22	32		4	
P3_10	67	M	2	367	196	22	19	15		3	
P3_11	59	M	2	1189	191	14	26	21		2	
P3_13	63	M	2	715	188	14	24	31		2	
P3_14	73	M	2	250	180	4	26	44		1	
P3_16	61	W	2	352	172	1	22	10		1	
P3_17	85	W	2	418	177	5	20	17		2	
P3_19	57	M	2	367	180	8	25	29		1	
P3_02	57	M	3	1175	185	8	18	69		5	
P3_07	80	W	3	475	170	8	23	50		6	
P3_12	59	W	3	712	170	5	18	39		5	

[1] Participants from the first cohort are identified by the prefix P2_, and participants from the second cohort by the prefix P3_

[2] Postural instability and gait difficulty score (PIGDs-Score) was calculated by the sum of the UPDRS 3 subscores of gait, postural stability and freezing.

Supplementary Table 2: Average stride length and heel strike angle (HSA) per walk

Participant ID[1]	Average SL ON-Med. [cm]			Average SL OFF-Med. [cm]		Average HSA ON-Med. [deg]			Average HSA OFF-Med. [deg]		Subjective Rating [VAS]		
	CT	FB	RV	CT	FB	CT	FB	RV	CT	FB	FB OFF	FB ON.	RV
P2_06	130,3	131,41		131,09	131,85	32,3	35,07		34,79	35,15	6	6	
P2_12	151,02	151,26		144,14	147,03	31,02	31,27		33,43	34,01	6	6	
P2_02	144,99	144,52		139,87	141,85	34,04	34,91		31,81	34,39	9	9	
P2_07	137,5	144,84		135,43	143,16	29,59	31,92		30,08	32,45	6	6	
P2_08	139,19	143,71		143,94	148,56	29,17	31,71		34,16	37,26	7	7	
P2_09	132,57	132,45		125,59	132,64	38,16	37,25		34,26	36,96	2	2	
P2_11	134,44	140,58		126,99	140,52	29,71	30,99		26,68	30,41	10	10	
P2_15	134,79	143,42		125,22	129,1	24,24	29,31		23,79	24,93	10	10	
P2_03	128,17	139		121,89	127,69	20,49	23,73		12,17	14,95	6	6	
P2_04	132,89	135,83		104,61	106,55	23,08	23,64		14,69	14,74	5	5	
P2_05	113,88	118,11		104,37	107,57	17,46	19,03		9,17	10,21	10	10	
P2_10	127,24	137,73		130,66	129,45	20,59	22,33		22,22	21,78	8	8	
P2_14	149,24	151,66		138,12	145,18	32,69	33,56		29,06	27,16	10	9	
P2_01	-/-	-/-		112,71	109,92	-/-	-/-		20,75	16,54	6	-/-	
P3_01	145,18	151,04	156,83			31,2	31,86	37,34				5	5
P3_09	170,52	167,13	171,93			35,79	36,22	36,82				3	4
P3_15	139,03	136,95	140,11			35,08	34,31	35,17				3	3
P3_20	122,89	124,25	122,14			31,33	32,21	30,03				7	7
P3_03	119,31	128,17	129,48			16,29	21,08	21,68				6	8
P3_04	142,79	145,06	139,39			36,1	36,69	34,43				5	4
P3_05	131,7	140,64	138,75			30,32	32,98	32,71				5	5
P3_06	134,99	134,13	130,51			26,25	25,96	23,66				6	4
P3_08	128,4	135,22	129,77			31,68	33,75	31,58				6	1
P3_10	151,93	151,23	145,44			28,24	27,66	25,94				9	9
P3_11	124,46	133,86	134,46			21,15	27,61	28,71				5	5
P3_13	158,88	161,27	157,85			36,56	36,51	36,58				3	3
P3_14	131,18	134,29	134,24			25,16	25,96	25,93				8	8
P3_16	127,92	131,77	131,14			26,73	28,2	26,32				8	6
P3_17	105,47	113,57	106,88			18,97	21,55	20,18				7	7
P3_19	143,43	142,64	143,37			35,7	34,19	35,49				8	9
P3_02	123,21	122,51	123,08			23,31	23,07	23,12				7	6
P3_07	106,55	106,57	107,12			17,54	18,13	19,08				6	5
P3_12	110,88	112,37	111,47			22,9	23,07	22,62				6	7

[1] Participants from the first cohort are identified by the prefix P2_, and participants from the second cohort by the prefix P3_

Supplementary Table 3: Video-labeled situations occurring during the walking course

Forward walking
Upward stairs
Downward stairs
Left Curve
Right Curve
Left Rotation
Right Rotation
Uphill walking
Downhill walking
Decelerating
Accelerating
Sitting down
Sitting
Standing up
Rotate left slowing down
Rotate right slowing down
Standing
Other

Supplementary Table 4: LMM – Stride length [cm]

Parameter	Estimate	Standard error	t-value	Degrees of freedom	p
(Intercept)	10.9	57.1	0.19	28	0.85
Height [cm]	0.818	0.33	2.49	27.8	**0.019**
Trial number	0.922	0.572	1.61	63.1	0.112
Second day of measurement	-1.38	1.31	1.05	63.8	0.298
Stimulation Mode * Medication State * H&Y stage<br>Stimulation Mode * Cohort	See marginal contrasts below				

Variable	Contrast	Difference	Standard error	Z-ratio	p
H&Y stages (in CT)	1-3	22.6	6.45	3.5	**<0.001**
	1-2	14.4	6.06	2.37	**0.018**
	2-3	8.23	5.66	1.46	0.146
Medication States (in CT)	ON Med.-OFF Med.	5.42	1.55	3.49	**0.001**
FB	FB-CT	3.44	0.82	4.2	**<0.001**
	RV-CT (in ON Med.)	2.06	1.32	1.56	0.118
	FB-RV (in ON Med.)	-0.63	1.34	-0.47	0.64

Effect	Interaction	Difference	Standard error	Z-ratio	p
ON Med.-OFF Med.	H&Y stage 1-3	-8.91	4.67	-1.908	0.056
ON Med.-OFF Med.	H&Y stage 1-2	-1.689	4.13	-0.409	0.682
ON Med.-OFF Med.	H&Y stage 2-3	-7.22	3.28	-2.2	**0.028**
FB-CT	ON Med.-OFF Med.	0.077	2.15	0.036	0.972
FB-CT	H&Y stage 1-3	-2.28	2.51	-0.01	0.363
FB-CT	H&Y stage 1-2	-3.96	2.22	-1.79	0.074
FB-CT	H&Y stage 2-3	1.68	1.95	0.863	0.388
FB-CT	Cohort 1 – Cohort 2	-1.83	2.03	0.902	0.367

Supplementary Table 5: LMM – Heel strike angle [deg]

Parameter	Estimate	Standard error	t-value	Degrees of freedom	p
(Intercept)	36.5	3.81	9.58	16.3	**<0.001**
Trial number	0.666	0.252	2.64	63.3	**0.0105**
Second day of measurement	-1.53	0.579	2.63	64.1	**0.0106**
Stimulation Mode * Medication State * H&Y stage<br>Stimulation Mode * Cohort	See marginal contrasts below				

Variable	Contrast	Difference	Standard error	Z-ratio	p
H&Y stages (in CT)	1-3	12.8	2.64	4.83	**<0.001**
	1-2	4.66	2.31	2.02	**0.043**
	2-3	8.11	2.08	3.9	**<0.001**
Medication States (in CT)	ON Med.-OFF Med.	1.07	0.69	1.57	0.117
Stimulation Modes	FB-CT	1.15	0.36	-3.18	**0.001**
	RV-CT (in ON Med.)	1.06	0.58	1.83	0.068
	FB-RV (in ON Med.)	-0.16	0.59	-0.28	0.780

Effect	Interaction	Difference	Standard error	Z-ratio	p
ON Med.-OFF Med.	H&Y stage 1-3	-9.12	2.06	-4.43	**<0.001**
ON Med.-OFF Med.	H&Y stage 1-2	-3.43	1.82	-1.89	0.059
ON Med.-OFF Med.	H&Y stage 2-3	-5.68	1.44	-3.94	**<0.001**
FB-CT	ON Med.-OFF Med.	0.349	0.95	0.367	0.714
FB-CT	H&Y stage 1-3	-0.184	1.11	-0.166	0.868
FB-CT	H&Y stage 1-2	-1.24	0.979	-1.26	0.207
FB-CT	H&Y stage 2-3	1.42	0.861	1.65	0.1
FB-CT	Cohort 1 – Cohort 2	-0.907	0.896	-1.01	0.311

Supplementary Table 6: LMM – COV of stride length

Parameter	Estimate	Standard error	t-value	Degrees of freedom	p
(Intercept)	1.93	0.071	27.1	8.9	**<0.001**
	0.015	0.016	0.985	63.8	0.328
	0.037	0.035	1.04	67	0.303
Stimulation Mode * Medication State*H&Y Stage<br>Stimulation Mode * Cohort	See marginal contrasts below				

Variable	Contrast	Difference	Standard error	Z-ratio	p
H&Y stages (in CT)	1-3	-0.24	0.08	-3.11	**0.002**
	1-2	-0.1	0.07	-1.49	0.135
	2-3	-0.14	0.06	-2.3	**0.022**
Medication States (in CT)	ON Med.-OFF Med.	-0.11	0.04	-2.48	**0.013**
Stimulation Modes	FB-CT	-0.004	0.02	-0.19	0.848
	RV-CT (in ON Med.)	0.02	0.04	0.43	0.67
	FB-RV (in ON Med.)	-0.06	0.04	-1.54	0.123

Effect	Interaction	Difference	Standard error	Z-ratio	p
ON Med.-OFF Med.	H&Y stage 1-3	0.168	0.123	1.36	0.1735
ON Med.-OFF Med.	H&Y stage 1-2	0.101	0.11	0.925	0.355
ON Med.-OFF Med.	H&Y stage 2-3	0.067	0.087	0.766	0.444
FB-CT	ON Med.-OFF Med.	0.043	0.059	0.730	0.465
FB-CT	H&Y stage 1-3	-0.073	0.068	1.07	0.287
FB-CT	H&Y stage 1-2	-0.008	0.061	-0.13	0.897
FB-CT	H&Y stage 2-3	-0.065	0.053	1.21	0.223
FB-CT	Cohort 1 – Cohort 2	0.09	0.056	1.62	0.105

Supplementary Table 7: LMM – COV of heel strike angle

Parameter	Estimate	Standard error	t-value	Degrees of freedom	p
(Intercept)	2.95	0.115	25.6	14.8	**<0.001**
Trial number	-0.053	0.021	2.51	63.7	**0.014**
Second day	0.078	0.048	1.61	65.5	0.113
Stimulation Mode * Medication State*H&Y Stage<br>Stimulation Mode * Cohort	See marginal contrasts below				

Variable	Contrast	Difference	Standard error	Z-ratio	p
H&Y stages (in CT)	1-3	-0.59	0.14	-4.15	**<0.001**
	1-2	-0.19	0.13	-1.5	0.134
	2-3	-0.41	0.11	-3.61	**<0.001**
Medication States (in CT)	ON Med.-OFF Med.	-0.11	0.06	-1.9	0.058
Stimulation Modes	FB-CT	-0.06	0.03	-2.13	**0.033**
	RV-CT (in ON Med.)	-0.04	0.05	-0.8	0.423
	FB-RV (in ON Med.)	-0.05	0.05	-0.95	0.343

Effect	Interaction	Difference	Standard error	Z-ratio	p
ON Med.-OFF Med.	H&Y stage 1-3	0.466	0.17	2.74	**0.006**
ON Med.-OFF Med.	H&Y stage 1-2	0.156	0.151	1.034	0.301
ON Med.-OFF Med.	H&Y stage 2-3	0.31	0.12	2.59	**0.01**
FB-CT	ON Med.-OFF Med.	-0.009	0.08	-0.115	0.909
FB-CT	H&Y stage 1-3	-0.025	0.093	-0.276	0.782
FB-CT	H&Y stage 1-2	0.017	0.082	0.204	0.838
FB-CT	H&Y stage 2-3	-0.042	0.072	-0.587	0.557
FB-CT	Cohort 1 – Cohort 2	-0.037	0.075	0.492	0.623
